# Economics of Task‐Shifting in Surgery: A Systematic Review

**DOI:** 10.1002/hsr2.71198

**Published:** 2025-09-03

**Authors:** Christian Inya Oko, Babar Ali, Mark Monahan, Abdullahi Tunde Aborode, John Okon, Francis Ayomoh, Chidiebube Ugwu, Onyeka Ekwebene, Shivangi Oza, Amada Ibe, Martilord Ifeanyichi, Calvin R. Wei, Aymar Akilimali

**Affiliations:** ^1^ University of Lancaster Bailrigg UK; ^2^ The University of Lahore Lahore Punjab Pakistan; ^3^ University of Birmingham Birmingham UK; ^4^ Department of Research and Development Healthy Africans Platform Ibadan Nigeria; ^5^ University of Oxford Oxford UK; ^6^ Department of Medicine Jefferson Einstein Philadelphia Hospital Philadelphia Pennsylvania USA; ^7^ College of Public Health East Tennessee State University Johnson City Tennessee USA; ^8^ Department of Public Health New York University New York New York USA; ^9^ University of Wolverhampton Wolverhampton UK; ^10^ London School of Economics London UK; ^11^ Department of Research and Development Shing Huei Group Taipei Taiwan; ^12^ Department of Research Medical Research Circle (MedReC) Bukavu Democratic Republic of the Congo

**Keywords:** cost benefit analysis, delegation, economic evaluation, task shifting, task‐sharing

## Abstract

**Background and Aim:**

Due to the global shortage in the surgical workforce, especially in low‐resource settings, one solution to increase surgical volume is to delegate certain roles of surgeons to other trained non‐surgeon health workers. However, quantifying the costs and benefits of surgical task‐shifting has several challenges associated with it. The purpose of this study was to conduct a critical appraisal of studies on the cost‐effectiveness of task shifting in surgical care.

**Methods:**

A systematic review was done using searches on four major electronic bibliographic databases (EMBASE, Ovid MEDLINE(R), Web of Science, Econlit) up to June 2021. Studies were selected based on pre‐defined inclusion criteria and relevant data were extracted.

**Results:**

A total of 16 studies were eligible for inclusion in the review. 14 of them were done in low‐ and middle‐income countries while the other two, in high income countries. Findings showed that task shifting to non‐surgeons lowers the total cost of surgery and increases coverage without any significant difference in outcome when compared with surgeons.

**Conclusion:**

Task shifting in surgical care is considered to be cost‐effective, improving the efficiency and access to surgical care in both low‐ and high‐income countries. Methodological challenges make study findings difficult to generalize. The costs and outcome values are dependent on the choice of comparator, hospital setting, cost items, and surgical procedure included. However, there is a need for more published data in different locations to support evidence for policymaking.

## Summary

- The purpose of this study was to conduct a critical appraisal of studies on the cost‐effectiveness of task shifting in surgical care.
- Task shifting in surgical care is considered to be cost‐effective, improving the efficiency and access to surgical care in both low‐ and high‐income countries. Methodological challenges make study findings difficult to generalize.
- The costs and outcome values are dependent on the choice of comparator, hospital setting, cost items, and surgical procedure included.

## 1 Introduction

The Lancet Commission on Global Surgery reported in 2015, that about five billion people in the world lack access to safe and affordable surgical care when needed [1, 2]. This is largely made up of those living in low and middle‐income countries (LMICs), where nine in ten people who need surgery cannot access it [1, 3]. Without measures for improvement in surgical care, LMICs will have a continual loss (US $12.3 trillion, with 2010US$ purchasing power parity) between 2015 and 2030 in their economic productivity [1, 4, 5].

Task‐shifting involves the rational redistribution of tasks, with specific roles delegated to health workers with less training and qualification to make the best use of the available human resources [6]. In surgery, task shifting has been shown to increase access to surgical care [7, 8, 9] and reduce its costs [10, 11, 12]. For instance, these tasks may include performing Cesarean sections, hernia repairs, appendectomies, or wound debridement by nonphysician clinicians such as clinical officers or assistant medical officers [8, 9, 10]. On the other hand, it has been noted to increase the risk of surgical complications as the lower cadres of health workers were considered to be less qualified and overworked with poor supervision [13, 14, 15, 16]. For decision‐makers in resource‐constrained environments, it is vital that they know whether an intervention such as task‐shifting represents a worthwhile investment in health spending [16].

An economic evaluation compares the costs and outcomes across two or more courses of actions [17]. Direct cost includes healthcare service cost while indirect costs are those that are not directly related to patient care. Evaluating the economics of task‐shifting in surgery has several challenges. First, the choice of comparator (task‐shifter against task‐shiftee) is an important consideration in determining its cost‐effectiveness. For clarity, task‐shifters refer to those health professionals from whom tasks are moved from (e.g., surgeons), while task‐shiftees are those onto whom the tasks are moved (e.g., non‐surgeon clinicians such as clinical officers or assistant medical officers) [6]. The choice of surgeon and task‐shiftee may be an unfair comparison considering instances where patients in rural areas would not have the option of a surgeon‐led operation in normal circumstances [18]. Second, there is an opportunity cost of retraining existing medical doctors to do surgery that should be captured to assess the full costs and consequences of task‐shifting [19]. This reduction in other clinical hospital duties to perform surgery is an important activity to quantify. Third, the additional surgery performed by the task‐shiftee may be subject to hospital constraints (e.g., number of different operating staff, theaters, hospital beds, supervision requirements) that can make the results of single center studies difficult to generalize [20, 21]. Finally, the focus on postoperative complications necessitates a comprehensive post‐discharge surveillance to estimate all relevant costs and consequences of task‐shifting [22, 23, 24, 25, 26].

While several reviews have explored the clinical effectiveness or safety of task‐shifting in surgical care, few have focused specifically on the economic dimension of this strategy. Moreover, studies that assess cost‐effectiveness often differ widely in methods, scope, and health system context [4, 5, 7, 8, 10, 15, 17, 26]. This review seeks to address that gap by critically appraising existing economic evaluations of surgical task‐shifting across different income settings.

The objective of this study is to critically appraise and assess economic studies that focus on task‐shifting for surgery. The studies will be assessed to identify the current state of the economic evidence base, and the differences in methodological approaches in assessing the costs and effectiveness of task‐shifting in surgery.

## 2 Methods

### 2.1 Search Strategy, Study Registration and Ethical Considerations

The literature search was done according to the center for review and dissemination (CRD) guideline [27] and was reported following the Preferred Reporting Items for Systematic Reviews and Meta‐Analysis (PRISMA) guideline [28]. A systematic and comprehensive search was done on four electronic bibliographic databases: Ovid MEDLINE(R), EMBASE, Web of Science (WoS) and Econlit from July 2000 to July 2021. The year 2000 was selected as the starting point because there was a lack of peer‐reviewed economic evaluations on surgical task‐shifting before this period. In addition, the early 2000s marked the emergence of more methodologically consistent and policy‐relevant studies, following key developments such as the WHO‐CHOICE initiative (launched in 1998) [12]. This allowed us to focus on literature aligned with modern standards of health economic evaluation. A representative search strategy included terms such as: “task shifting,” “economic evaluation,” “surgical care,” “clinical officer,” and “cost‐effectiveness.” Additional references were retrieved from the references of selected articles. This study protocol was registered in the International Prospective Register of Systematic Review (PROSPERO) database (registration number: CRD420254600901). As this is a systematic review based on secondary data, ethical approval and informed consent were not required.

### 2.2 Eligibility Criteria

#### 2.2.1 Inclusion Criteria

- Studies were included if they evaluated the cost and/or outcomes of surgical task shifting, specifically involving the full delegation of tasks from specialist surgeons (task‐shifters) to less trained/qualified health workers (task‐shiftees), across any income setting. The cost values could be borne by the patients, healthcare providers or the society.
- Studies evaluating full or partial economic evaluations (cost‐effectiveness, cost–benefit, or cost analysis) in high‐, middle‐, and low‐income settings.
- Peer‐reviewed articles published in English between January 2000 and July 2021.

#### 2.2.2 Exclusion Criteria

- Studies solely focused on clinical outcomes without economic analysis
- Studies that focused solely on task sharing—where roles are jointly performed—were excluded unless the task‐shifting component could be clearly isolated and economically assessed.
- Editorials, conference proceedings, protocols, commentaries, and papers not published in English language.

### 2.3 Study Selection, Data Extraction, Analysis, and Reporting

**Figure 1:**
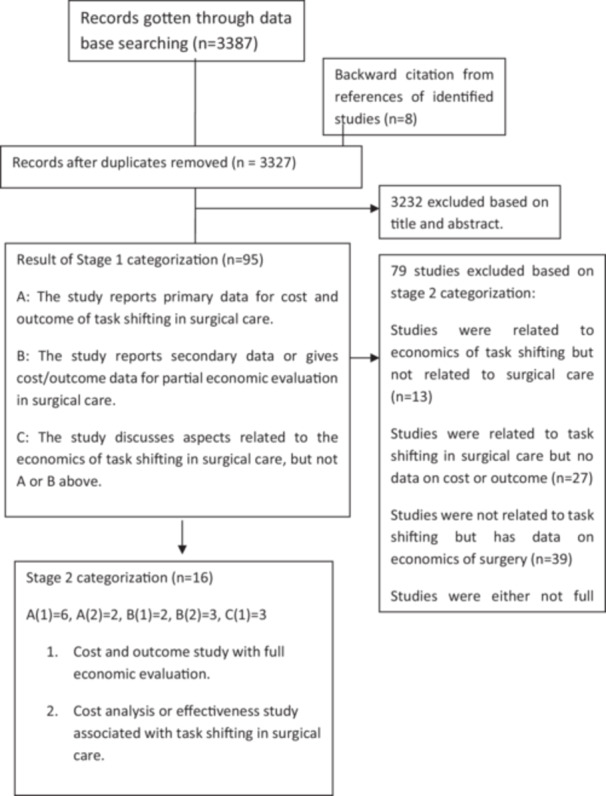
Prisma chart.

The PRISMA chart (Figure 1) provides a step‐by‐step representation of the systematic review process, starting with the identification of 3387 records through combined searches across four databases (Ovid MEDLINE, EMBASE, Web of Science, and EconLit), followed by eight additional records obtained via backward citation tracking. The number of hits from each individual database was not recorded separately. After removing duplicates, 3327 records were screened based on title and abstract, leading to the exclusion of 3232 records. In the second stage of categorization, 79 studies were excluded for not meeting the inclusion criteria, leaving 16 studies for the final analysis. This diagram ensures transparency and reproducibility of the study selection process by detailing every stage of inclusion and exclusion. Studies relevant to the review were selected in a two‐stage categorization process [29]. Their titles and abstracts were initially screened against the eligible criteria, then the full papers of potentially relevant articles were read and further classified. One investigator (CO) carried out all study screening and data extraction while another investigator (AI) undertook the screening of a random 30% to assess agreement. Discordances were resolved through discussion, and a third investigator (MM) was sought where agreement could not be readily reached. The data extracted were tabulated and compared across the individual studies using a process of narrative synthesis. To improve comparability of cost findings, different cost values were converted to international dollars and adjusted by their country's Purchasing Power Parity (PPP) conversion factor [30]. In cases where a country did not have a PPP conversion factor, an implied PPP conversion factor from the IMF was used instead [31]. For inflation purposes, studies without a specified cost year were assumed to be the last year of data collection.

## 3 Results

The results of this systematic review provide a comprehensive evaluation of task‐shifting in surgical care, focusing on its economic implications, training structures, and outcomes. Sixteen studies meeting the inclusion criteria were analyzed, encompassing diverse healthcare settings in low‐, middle‐, and high‐income countries. The studies highlighted substantial variations in task‐shifting practices, including the roles and training of task‐shiftees, cost components considered, and outcome measures assessed. This section presents the findings under key themes, including the general characteristics of the studies, cost analysis, and cost‐effectiveness evaluations, providing critical insights into the economic and practical impact of surgical task‐shifting.

### 3.1 General Study Characteristics

Task‐shifting roles and training requirements showed notable diversity across countries, reflecting differences in healthcare needs and resource availability. According to classifications by World Bank data [32], nine of the studies were conducted in low‐income countries (LICs) (Burkina Faso (*n* = 1), Ethiopia (*n* = 1), Malawi (*n* = 8), Mozambique (*n* = 2), two in lower middle income countries (LMICs) (Tanzania (*n* = 1), Senegal (*n* = 1) and two in a high‐income countries (HICs) (United States of America (*n* = 2). All studies were done in single centers with majority of studies were set in rural hospitals and rural healthcare training centers [24, 33, 34] while two studies were in urban areas [35, 36]. The surgical procedure types comprised obstetric surgeries (*n* = 7) [18, 24, 33, 34, 37, 38], orthopedic surgeries (*n* = 3) [22, 23, 24], general surgeries (*n* = 3) [26, 39, 40], cardiac surgery (*n* = 1) [35], maxillofacial surgery (*n* = 1) (OMS) [36], and pediatric surgery (*n* = 1) [41] (Table 1).

**Table 1:** Task shift and description in different countries.

Lead author (year)	Country	Task‐shifter comparator (training)	Task‐shiftee (training)	Study description
Kruk (2007) [38]	Mozambique	Surgeon (Obstetrics Specialist)	AMO (5 years)	Mozambique's surgically trained AMOs demonstrate cost efficiency in major obstetric surgery compared to specialist physicians, with $38.9 per surgery versus $144.1, maintaining similar surgery reasons and complication rates.
Hounton (2009) [18]	Burkina Faso	GP (post graduate training)	CO (3 years diploma, 1 year internship)	In Burkina Faso, comparing Cesarean sections, obstetricians exhibited 10% faster procedures and 30% shorter postoperative stays, likely due to their advanced obstetric skills, enhancing effectiveness.
Grimes (2014) [22]	Malawi	MO (undergraduate medical trainings)	AMO (5 years)	Assessing Malawi's Orthopedic Clinical Officer (OCO) training, the study shows $92.06 per DALY averted. Crucial for all ages, especially children and workers, it advocates nonphysician clinicians to enhance low‐resource orthopedic access.
Mkandawire (2008) [23]	Malawi	MO (undergraduate medical trainings)	AMO (5 years)	Malawi's Orthopedic Clinical Officer Program trains specialized personnel to address orthopedic care scarcity, empowering them to manage rural hospitals' workload effectively, with 117 officers trained and 82 active practitioners noted.
Resnick (2016) [41]	United States	—	Physician assistant (‐)	Integrating physician assistants (PAs) into oral and maxillofacial surgery (OMS) reduced costs by $75.08 per procedure, enhanced efficiency, and maintained safety. PAs increased patient throughput, implying improved practice effectiveness and affordability.
Nabagiez (2016) [40]	United States	—	Physician assistant	Assessing a physician assistant home care program post‐cardiac surgery, the study revealed a 41% reduction in 30‐day readmissions, saving $977,500 in 2 years. Home visits by PA emerged as cost‐effective for curbing cardiac surgery readmissions.
Pereira (2011) [25]	Tanzania	MO (undergraduate medical trainings)	AMO (5 years)	Addressing Tanzania's healthcare context, the study underscores assistant medical officers (AMOs) as pivotal in emergency obstetric surgeries due to doctor scarcity. Despite a modest case fatality rate, achieving full met need for emergency obstetric care remains below the 100% target.
Gessessew (2011) [33]	Ethiopia	—	NPC	Examining NPC utilization in Tigray, Ethiopia, the article shows NPCs performing substantial obstetric procedures, mirroring physician outcomes. Strengthening NPC training for emergency obstetric surgery is urged to combat high maternal mortality.
Chilopora (2007) [34]	Malawi	MO (Undergraduate Medical trainings	CO (3 years, diploma, 1 year internship)	Malawi's clinical officers conducted 90% of simple Cesarean sections, yielding outcomes akin to medical officers. This underscores clinical officers' vital role in maternal and neonatal care, essential due to physician scarcity. The study assessed maternal and neonatal conditions, pyrexia, wound matters, reoperation necessity, neonatal results, and maternal mortality.
Periera (1996) [24]	Mozambique	MO (Undergraduate Medical trainings)	AMO (5 years)	This study found that the AMOs, compared to MOs, did 92% of the surgeries in district hospitals, 53% in Central hospitals, and 34% in general/provincial hospitals. They were also found to stay longer in rural areas. After 7 years, around 90% of AMOs were still working in district hospitals, while almost no MO remained there.
Brouwere (2009) [37]	Senegal	Anesthetist, GP and surgical assistant (basic medical trainings)	Obstetrician (specialty training)	Senegal's study on task shifting for emergency obstetric surgery in district hospitals reveals increased c‐section rates and newborn outcomes through redistributing tasks to less specialized workers. However, inconsistent effects, linked to surgical team availability variations, prompt examination of influencing factors and policy implications for Senegal and similar contexts.
Wilhelm (2017) [35]	Malawi	Surgeon (orthopedic/general surgery specialists)	Orthopedic CO (18 months diploma, with 4 years working experience)	Examining Malawi's nonphysician clinicians in orthopedic surgery, the study shows positive outcomes and minimal complications. The authors suggest formal training and standardized protocols to enhance practice quality.
Wilhelm (2011) [26]	Malawi	—	CO (3 years diploma, 1 year internship)	Evaluating Malawi's COs in major general surgery, the study confirms their safety and efficacy with adequate training and supervision. Outcomes matched surgeon‐performed procedures.
Tyson (2014) [42]	Malawi	Physician (consultant surgeon or surgical resident)	CO (3 years diploma, 1 year internship)	—
Gajewski (2018) [39]	Malawi	MO (undergraduate medical trainings)	CO (3 years, diploma, 1 year internship)	The study compares hernia surgery outcomes in district and central hospitals in Malawi, finding no significant differences. District hospitals can meet rural surgical needs with investment in training and resources, supervised by higher‐level institutions.
Beard (2014) [36]	Tanzania	Physician	NPC	The study in Tanzania reveals NPCs can perform major surgeries effectively, offering a solution for the surgical workforce crisis in low‐resource settings. Challenges include ensuring safety and establishing clear roles and training systems.

Abbreviations: AMO, assistant medical officer; CO, clinical officer; GP, general practitioner; MO, medical officer; NPC, nonphysician clinicians.

The comparator used in the studies for the task‐shiftee included surgeons for the different surgical specialties (*n* = 3) and non‐surgeons (*n* = 8) [24, 33, 34, 35, 36, 37, 38, 39] and a nonsurgical condition (HIV care) [22]. Three studies did not report a comparator. The absence of a comparator limits the ability to assess the relative cost‐effectiveness of task‐shifting, as it removes a benchmark against which to measure outcomes or cost savings. The definition of and the level of qualifications needed for the task‐shiftee varied across studies. The task‐shiftee was called a CO (*n* = 5), Orthopedic CO (*n* = 1), AMO (*n* = 5), NPC (*n* = 1), Physician Assistant (*n* = 2), and Obstetrician etc [24, 33–39]. Four studies did not require a medical qualification for their task‐shiftees. In the LICs and LMICs, the task‐shiftees task was to perform surgical procedures. On the other hand, the task‐shiftee physician assistants in the HICs were only assistants in cardiac surgery and OMS [35, 36]. However, two studies [18, 37] broadened the scope of task shiftees tasks to include the medically qualified General Practitioner (GP) (Table 1).

### 3.2 Cost Analysis

Cost components varied across the studies in surgical task‐shifting, with some including training costs while others focused on deployment or procedural costs. For example, training costs were more thoroughly detailed in studies from Malawi and Mozambique, with some studies including indirect costs such as opportunity costs of training. Of the cost studies, 4 out of 6 studies considered the task‐shiftee training costs. They include training, deployment and procedure costs. Two studies [22, 23] reported the cost of training orthopedic clinical officers (OCO) once over 18 months; whereas, another [18] considered costs for ongoing periodic training of task‐shiftees. Inclusion of the training cost for task‐shifters and task‐shiftees was done by only two studies (18, 35). One study [38], gave the cost of training an obstetrician as 30% higher than that of a medical officer and 80% greater when compared with a clinical officer. However, a second study (18) reported a 30% increase in training a medical officer for 6 years, compared to an assistant medical officer for only 3 years. Only 2 studies [22, 38] considered the opportunity cost for task shiftees in training. This was taken to be about 80% of their salaries [38] with no justification given on the value used (Table 2).

**Table 2:** Studies with included costs.

Author (year)	Cost components	Cost data source	Data collection period	Year of cost/currency	Discount of costs
Kruk (2007) [38]	Task‐shiftee training; pre and post start up; deployment	Review of budgets, annual expenditure reports, enrollment registers, accounting statements interviews	30 years	2006 US Dollar	3%
Grimes (2014) [22]	Task‐shiftee training; deployment: Surgical common operation equipment	Hospital accounting statements; local hospital pharmacy store; WHO CHOICE website	6 months	2012 US Dollars	N/A
Hounton (2009) [18]	Training; deployment	Hospital accounting statements, University training log book, Ministry of health medical officers log book.	30 years	2006 CFA	3%
Mkandawire (2008) [23]	Training: Salaries of two orthopedic clinical officer instructors, books, basket of orthopedic tools	Hospital log book	6 months	2012 US Dollar	N/A
Resnick (2016) [41]	Median salaries fringe benefits overhead cost	US Bureau of Labor Statistics, American Dental Association, American Academy of Physician Assistants, National Society of Certified Healthcare Business Consultants.	30 days	2016 US Dollar	N/A
Nabagiez (2016) [40]	Patient visit time; travel expenses; salaries	Hospital bill	4 years	2016 US Dollar	N/A

Deployment cost refers the financial value of assigning skilled workers to work in the different places where they are posted [42]. This includes salaries, fringe benefits and overhead cost associated with the practice. Four studies [18, 22, 36, 38] considered the opportunity cost of deployment. For obstetrics surgery, two studies [18, 38] gave the cost of specialist task‐shifters as three times that of nonspecialist task‐shiftees (AMO). However, only the deployment cost due to task‐shiftees was stated by the other two studies [22, 36]. Also, a study conducted by Mkandawire et al. [23] showed that after 7 years, none of the orthopedic surgeons remained in the rural hospitals after training compared to the OCO that were all retained in rural clinical practice. Thus, Kruk et al. [38] recommended rural allowances and annual tips for case referrals to be given to encourage non surgeons working in rural hospitals (Tables 1 and 2).

The cost due to surgical procedures includes the salaries, consumables, overheads and any postoperative complications arising from the operation. The differential cost of different types of surgical equipment used by the two cadres based on their skill was not indicated. However, only one study [22] considered the procedure costs for task‐shiftee. The costs that are included in the studies depended on their stated perspective. Out of the six cost studies, three [18, 22, 36] were by the hospital/provider perspective, while one study [38] widened the scope to a modified societal perspective (Tables 1 and 2).

### 3.3 Outcome

Outcome from surgical task‐shifting varied across studies. Work output (*n* = 8), patient outcome (*n* = 6), and time duration (*n* = 1) were outcomes of interest across studies. Eight studies [23, 24, 25, 26, 34, 35, 36, 37, 38, 39] compared the surgical work output as a measure of outcome. All of the studies reported a higher proportion of surgeries done by the task‐shiftees in the district hospitals, compared to the task‐shifters. However, two studies [26, 39] stated that while both cadres performed an equal number of major surgeries, the task‐shiftees did more of the minor surgeries in the rural hospitals than the surgeons. One study [36] compared the outcome with the time it took to do molar extraction procedure in oral and maxillofacial surgeries, with or without a physician assistant (PA). It was noted that whereas it took the surgeon 37.6 min to do an extraction without the PA, it rather took 29.2 min for the same procedure when a PA is available to assist, with no statistically significant difference in postoperative complications.

Patient peri‐operative outcome was evaluated by four studies [26, 39, 40, 41]. There was found no statistically significant difference in the symptomatic outcome of operation done by task‐shiftees in district hospitals compared with surgeon task‐shifters in the central hospitals. However, one study [18] reported that the case fatality rate for the newborn after Cesarean section was higher for Clinical Officers compared with Surgeons. Generally, three studies [37, 39, 41] stated that the deployment of task‐shiftees in district hospitals increased access reducing the number of maternal and neonatal deaths. The neonatal death was noted to have been halved from 23.1% stillbirths per 100 Cesarean section deliveries to 12.1% in district hospitals. Only one study [22] reported outcome as Disability Adjusted Life Year (DALY). DALY is a measure of the disease burden, described as one lost year of healthy life. This consists of estimates of years of life lost through death (YLL) added to that lived with disability (YLDD) [17, 42]. These were used to calculate the total DALYs averted per hospital [22, 43].

### 3.4 Cost‐Effectiveness and Sensitivity Analysis

Studies evaluating cost‐effectiveness consistently demonstrated the economic advantage of task‐shifting in surgical care. Four out of sixteen studies [18, 22, 23, 38] analyzed the cost‐effectiveness of the intervention and comparator. One study [38] estimated the cost per major obstetric procedure done by obstetricians to be three times that for Assistant Medical Officer (AMO). The cost‐effectiveness value was $38.87 for AMO (task shiftee) compared to $144.1 for physician (comparator). The study by Hounton et al. [18] measured the cost for saving a newborn's life using ICER of cost per newborn death averted (x 1000 live birth). This gave $3235 for obstetrician rather than CO; and $200 for GP compared with CO. Although a threshold value of acceptability was not given, the ICER for the GP option was considered lowest. Mkandawire et al. [23] did not give clear values; it reported orthopedic surgery by OCO to be cost‐effective. Unlike other studies, Kruk et al. (2007), did a one‐way sensitivity analysis which retained a substantial cost advantage for AMO compared to Physicians [38]. These results underscore task‐shifting as a cost‐effective strategy to address surgical workforce shortages while maintaining high standards of care (Table 3).

**Table 3:** Cost effectiveness of full evaluation studies.

Author (year)	Study type	Outcome measure	Cost of comparator	Cost of intervention	Output of task‐shifter	Output of task‐shiftee intervention	Results
Kruk (2007) [38]	CEA	Cost per major surgery	$167,057.7	$71,914.8	117 surgeries/surgeon	102 surgeries/AMO	Cost per surgery: $38.87 for AMO $144.1 for physician
Hounton et al. (2009) [18]	CEA	Cost per averted new born death (ICER)	$513/CS	‐GP:$207/CS, CO: $193/CS	Newborn case fatality rate: 99/1000	‐GP newborn case fatality rate: 125/1000 ‐CO newborn case fatality rate: 198/1000	Cost per averted new born death (x 1000 live birth): $200 for GP rather than CO. $3235 for obstetrician rather than CO; $11,757 for Obstetrician rather than GP
Grimes et al. (2014) [22]	CUA	Cost per DALY	N/A	$169,144.86	N/A	N/A	CO: $92.06/DALY
Mkandawire et al. (2008) [23]	CEA	Cost per work output	$52,000	$7253	10%–20% in district hospitals	80%–90% in district hospitals	

Abbreviations: AMO, assistant medical officer; CO, clinical officer; MO, medical officer; NA, not available; NPC, nonphysician clinician.

## 4 Discussion

### 4.1 Statement of Principal Findings

The aim of this study is to critically appraise and assess economic studies in surgical task‐shifting. Surgical task‐shifting was reported to be cost‐effective with the task‐shiftees noted to have generally lower costs and similar outcomes to task‐shifters. However, the review found high heterogeneity in the assessment of costs and outcomes in surgical task‐shifting which merit caution on the literature findings. The foremost key methodological challenge is the comparator used in most studies. In the majority of cases, the comparator was from an urban setting. This contrasts with the rural setting of the task‐shiftees [39, 41]. This brings about an unequal standard of comparison as the intervention (task shiftee) may not have the same operating conditions nor patient profile as the comparator (surgeon) [16]. Other studies had used non‐surgeons (physicians or medical officers) as comparators. This leads to a different research question across studies in terms of comparing non‐surgeons to less qualified non‐surgeons or surgeons to non‐surgeons [42, 43].

Second, identification and measurement of all relevant costs is an important step in assessing the economic rationale of deploying surgical task‐shiftees. Yet, there was variable efforts incorporating key costs such as training costs, procedure costs, postoperative costs, and the opportunity cost of redeployment. For training costs, surgeon training costs were sometimes not included while the task‐shiftee's training costs were included making the total cost comparison is insufficient. Ongoing training costs including refresher courses were specified in only one study which may otherwise underestimate the costs associated with task‐shiftees. For procedure costs, this may be an important factor in the total cost difference since there may be different surgical instruments suitable for their varying skill levels. A skilled surgeon may choose to use a complex laparoscopic set while a clinical officer may rather cut open with a cheaper scalpel [39, 44].

For postoperative costs, the lack of postoperative discharge surveillance schemes may be undercounting the adverse outcomes and costs [45]. As task‐shiftees are usually associated with more adverse outcomes, non‐collection of this information may make task‐shifting appear more cost effective than is the case. For the opportunity cost of deployment, the lack of agreed values on quantifying these costs adds uncertainty on the true costs of the benefits forgone of physicians and surgeons deployed in typically rural areas. In the included studies, the opportunity cost of deployment was given to be three times greater for obstetricians than the assistant medical officer [18, 38]. This increased cost for the surgeons made it difficult to retain specialist doctors in rural communities to meet the surgical needs of the population. The recommendation for salary increases and rural allowances is an important consideration to encourage the stay of surgeons and improve the welfare of task‐shiftees in rural hospitals. This will likely improve their output and discourage brain drain.

Third, there are multiple outcomes that can be used when evaluating task‐shifting and restricting oneself to one natural outcome may mask other important outcomes especially when the outcomes do not all go in the same direction with task‐shifting. Thus, a cost consequence analysis may be appropriate where no relevant composite outcome can be used to provide decision makers with information on the consequences of task‐shifting. As mentioned previously, the setting may have an impact on outcomes where included studies reported that non‐surgeons did about 85% of the surgeries studied in rural hospitals with almost the same perioperative and mortality outcome as a comparator [45, 46, 47]. These included about half of all major orthopedic and general surgeries and almost all minor surgeries and obstetric emergencies. Therefore, it implies that in rural areas, this cadre of health workers (non‐surgeons) handled emergency surgical cases while some major cases were be referred to surgeons in the central hospitals according to their specialty.

However, the postoperative complication rates were suggested to be different in task‐shiftees. There was a marginally higher rate of newborn and maternal death in Cesarean deliveries done by clinical officers compared to the obstetricians. The cause of these deaths could be due to the lack of certain surgical skills that could save the lives of baby and mother or unavailability of necessary resuscitation facilities in rural hospitals [18, 37]. Solutions to these could be provided by a regular update training course for such cadres of health workers to improve their surgical skills. However, there should also be adopted guidelines that define limits for referral of cases to specialists [18, 23]. Promotions and an increase in salaries and welfare should also be offered to the health workers working in rural hospitals [37]. These will increase their efficiency and retain them in the rural district hospitals where they offer improved surgical care at a lower cost [48].

Task shifting was accounted to be cost‐effective intervention across all studies that did a full economic evaluation. The cost per percentage output remained low for non‐surgeons since they were based in rural district hospitals and had more patients to attend to. They reduced cost, had a marginal increase in death rate, and were three times more cost‐effective than the surgeons. This implies that it could be effectively applied in low‐income countries like Malawi as the cost per DALY averted was also lower than the per capita gross domestic product (GDP) per DALY averted [22].

There was a paucity of studies with full economic evaluations with the majority of the studies comparing the outcome of surgical task shifting. Also, the responsibilities for task‐shiftees were noticeably different in HICs versus LMICs. Studies that were done in high income countries only considered physician assistants who were trained to do minor tasks in surgical care while the surgeons took responsibilities for the major ones [49, 50]. This contrasted with the LMICs where the task shiftees took full charge of all tasks surgical care [24, 34, 35, 36, 37, 39]. This would probably contribute to better surgical care coverage in areas of low surgeon workforce [16]. Additionally, supervision structures differed notably between HICs and LMICs. Task‐shiftees in HICs generally operated under closer supervision, whereas in LMICs, they often performed independently due to workforce shortages. This difference may influence outcomes. Cultural expectations, health system structures, and regulatory frameworks also shape how task shifting is implemented across settings [37, 38, 39].

Across the included studies, the most commonly involved cadres in surgical task‐shifting were Clinical Officers, Assistant Medical Officers, and General Practitioners. These cadres often worked in government‐run or rural district hospitals, particularly in LMICs such as Malawi, Tanzania, and Mozambique. While the majority of studies reported no significant difference in outcomes, a few highlighted higher maternal or neonatal complications in specific procedures (e.g., Cesarean sections) when performed by task‐shiftees. These differences were often attributed to contextual factors such as lack of supervision, infrastructure gaps, and case selection [19, 22, 23, 24, 25, 26].

In addition, the number of comparators (surgeons) in the health systems was few while the task shiftees (non‐surgeons) had greater number based in the district hospitals where the majority of the population dwell [16, 18, 22]. Thus, task shifting helped to reach out surgical services to the large number of people living in the rural areas that may not have good access to the urban hospitals when on emergency [18, 38]. Therefore, the teaching hospitals must increase the training capacity for surgeons to enhance the number of skilled surgeons to handle complicated cases on referral [18, 23].

### 4.2 Strengths and Limitations of the Study

#### 4.2.1 Strengths

First, this study, to the best of our knowledge, is the first systematic review that has critically analyzed the economics of task shifting in surgical care as no earlier study was found on Cochrane. The search strategy was comprehensive with extensive searches done on four databases over 20 year period. In addition, the surgical procedures that were considered included both major and minor surgeries across the main surgical subspecialties. This shows the study covered a wide range of surgical interventions. Moreover all, income country groups were considered. This gives a global perspective with insight into the methodological considerations and the potential data gaps in assessment from the contrasting settings.

#### 4.2.2 Limitations

This is mainly the English language restriction used for the article inclusion criteria. This is due to the fact that there was no available resource to translate the non‐English papers. Shenderovich et al. [48] found that including only English language studies reduce the number of potentially relevant articles by about 15%. Also, the result of searches done gave studies done in Africa and the United States only. This poses limitation to the globalization of findings. In addition, the PPP exchange rate is used for adjustment of cost‐of‐living differences between countries. However, with this, there is the likelihood of introducing measurement error in the study findings [46, 47]. Another limitation was the inconsistency in how costs and outcomes were measured across studies. Different units of analysis, currency conversions, and outcome definitions limited direct comparability and introduced heterogeneity in the synthesis.

### 4.3 Comparison With Other Studies

Previous review studies have evaluated task‐shifting in surgical care [33, 34, 35, 36, 37, 38, 39, 40, 41, 42, 43, 44]. There have also been other reviews that assessed the cost of task‐shifting in other health care services [51, 52, 53, 54, 55, 56]. However, through the searches made, there was no available review study that analyzed different studies focused on the economics of surgical task‐shifting. With the global shortage in health workforce, there is need to assess the economic implication of task‐shifting in surgical care so as to inform health policy and improve global surgical care [57, 58].

#### 4.3.1 Implications for Policy and Practice

Task shifting policies in surgical care has proved to be cost‐effective and essential in meeting the needs of rural areas in countries like Malawi, Mozambique and Tanzanias [18, 23]. With efforts towards Global Surgery 2030, task shifting is considered as a viable strategy in strengthen the surgical workforce [59]. Its implementation would be an important intervention for improving surgical access for rural dwellers. However, the roles of the non‐surgeon must be clearly stated and limits placed to encourage timely referral of complicated cases to surgeons [59]. Policymakers should also ensure that the incentives due to non‐surgeons who take up surgical roles in the rural district hospitals are duly given to them, to encourage their stay there. Likewise, surgeons should also be given their due salaries and allowances to encourage them to continue to diligently discharge their duties.

## 5 Conclusion

Globally, the health workforce to meet the increasing surgical health needs of the population is inadequate, especially in LMICs. Task shifting from surgeons to non‐surgeons is a strategy that has been employed by some countries to improve the health workforce in surgical care. Overall, this study concludes that delegating procedures in surgical care to trained health workers of the lower cadres is cost‐effective across the high, lower‐middle‐ and low‐income settings and will increase access in rural areas. Update refresher courses and timely referral of complicated cases was found to improve perioperative outcome. Improvement in salary structure, allowances and other benefits were also noted to encourage stay of non‐surgeons in rural areas as well as surgeons in the country, particularly in LMICs. Finally, there is need for more published data in different geographical settings with standardized data collection method for cost and outcome, to support evidence for policymaking.

## Author Contributions

**Christian Inya Oko:** conceptualization, investigation, visualization, project administration, resources. **Babar Ali:** conceptualization, methodology, investigation, writing – original draft, writing – review and editing, formal analysis, data curation. **Mark Monahan:** resources, supervision, project administration, writing – review and editing. **Abdullahi Tunde Aborode:** conceptualization, methodology, software, data curation, supervision, formal analysis, validation, investigation. **John Okon:** funding acquisition, writing – original draft, visualization, writing – review and editing, project administration, resources, supervision. **Francis Ayomoh:** validation, funding acquisition, writing – original draft. **Chidiebube Ugwu:** conceptualization, validation, formal analysis, supervision, resources, project administration. **Onyeka Ekwebene:** software, formal analysis, project administration, data curation, supervision, resources. **Shivangi Oza:** investigation, validation, formal analysis, supervision. **Amada Ibe:** funding acquisition, visualization, writing – review and editing, writing – original draft, project administration, resources. **Martilord Ifeanyichi:** data curation, supervision, project administration, visualization. **Calvin R. Wei:** conceptualization, funding acquisition, writing – original draft, writing – review and editing. **Aymar Akilimali:** conceptualization, methodology, software, data curation, investigation, funding acquisition, writing – original draft, writing – review and editing, visualization, validation, formal analysis, project administration, supervision, resources.

## Consent

All authors have read and approved the final version of the manuscript. Aymar AKILIMALI had full access to all of the data in this study and takes complete responsibility for the integrity of the data and the accuracy of the data analysis. As this is a systematic review based on secondary data, no new data was generated and hence data shearing in not applicable.

## Conflicts of Interest

The authors declare no conflicts of interest.

## Transparency Statement

The lead author Aymar AKILIMALI affirms that this manuscript is an honest, accurate, and transparent account of the study being reported; that no important aspects of the study have been omitted; and that any discrepancies from the study as planned (and, if relevant, registered) have been explained.

## Data Availability

Data sharing not applicable to this article as no datasets were generated or analyzed during the current study. The authors confirm that the data supporting the findings of this study are available within the article. Data sharing is not applicable to this article as it is a systematic review.
